# Multidrug-Resistant Extended-Spectrum Beta-Lactamase (ESBL)-Producing *Escherichia coli* in a Dairy Herd: Distribution and Antimicrobial Resistance Profiles

**DOI:** 10.3390/antibiotics13030241

**Published:** 2024-03-05

**Authors:** Martina Penati, Laura Musa, Laura Filippone Pavesi, Alessandro Guaraglia, Fernando Ulloa, Paolo Moroni, Renata Piccinini, Maria Filippa Addis

**Affiliations:** 1 Department of Veterinary Medicine and Animal Sciences, University of Milan, 26900 Lodi, Italy; martina.penati@unimi.it (M.P.); laura.musa@unimi.it (L.M.); laura.filippone@unimi.it (L.F.P.); paolo.moroni@unimi.it (P.M.); renata.piccinini@unimi.it (R.P.); 2 Department of Humanities and Social Sciences, University of Sassari, 07100 Sassari, Italy; aguaraglia@uniss.it; 3 Escuela de Graduados, Facultad de Ciencias Veterinarias, Universidad Austral de Chile, Valdivia 5090000, Chile; fernando.ulloa@uach.cl; 4 Laboratory of Infectious Diseases of Domestic Animals (MiLab), University of Milan, 26900 Lodi, Italy

**Keywords:** antimicrobial resistance, biosecurity, dairy calves, ESBL *E. coli*, multidrug-resistant, waste milk

## Abstract

This study investigated the presence, distribution, and antimicrobial resistance profiles of extended-spectrum beta-lactamase (ESBL)-producing *Escherichia coli* in a dairy herd located in Northern Italy. The feces of clinically healthy calves, their mothers, and the cows treated for mastitis, as well as water, environmental samples, and waste milk were collected and subjected to bacteriological culture on CHROMagar^TM^ ESBL plates. A questionnaire was administered to identify risk factors. The isolates were identified as *E. coli* by MALDI-TOF MS and subjected to the double-disk synergy test (DDST) and minimal inhibitory concentration (MIC) assay. As a result, ESBL *E. coli* was isolated from the feces of 28 of 37 (75.67%) calves, the feces of 2 of 3 (66.67%) treated cows, 8 of 14 (57.15%) environmental samples, and waste milk. All ESBL isolates showed multiple resistances and were categorized as multidrug-resistant (MDR). Several risk factors for ESBL *E. coli* selection and diffusion were identified, including lack of routine cleaning of calf feeding and housing equipment, administration of waste milk to male calves, and blanket dry cow therapy. In conclusion, this study highlighted the presence of MDR, ESBL *E. coli* in the feces of most dairy calves, and their association with different sample sources. Accordingly, adding to the prudent use of antibiotics, the adoption of adequate farm hygiene and biosecurity measures might also help prevent the spread and transmission of ESBL *E. coli* within the herd.

## 1. Introduction

Antimicrobial resistance (AMR) is a problem of global concern [1]. The rise in antibiotic-resistant bacteria, as stated by the World Health Organization (WHO), can render many drugs previously essential for treating infections in humans and animals ineffective [2]. About 50–80% of the total antibiotic use in developed countries has been attributed to livestock [3], although significant efforts are being made to reduce antibiotic use. According to a report published by the European Food Safety Authority [4], in Europe antibiotic use has decreased to become lower in food-producing animals than in humans. Between 2016 and 2018, animals used less antimicrobials than humans overall. In 2017, for example, animals averaged 108.3 mg/kg (range 3.1–423.1) compared to 130.0 mg/kg (range 52.8–212.6) for humans [4]. Nevertheless, the animal industry still plays a crucial role in the occurrence and transmission of AMR [5].

In recent years, the prevalence of bacterial strains producing extended-spectrum beta-lactamases (ESBLs) has increased worldwide [6,7]. These enzymes are able to hydrolyze third-generation cephalosporins and aztreonam but are inhibited by clavulanic acid. ESBL-producing organisms often show co-resistance to many other classes of antibiotics. Adding to problems in veterinary medicine, this group of plasmid-mediated, rapidly evolving, and diverse enzymes poses major therapeutic challenges in human medicine, especially concerning the treatment of hospitalized and community-based patients [7].

ESBL-producing *Escherichia coli* is a common occurrence in dairy cattle, with the highest incidence observed in calves [8,9]. Food-producing animals acquire AMR microorganisms due to several factors such as antibiotic use, forage, soil, water [3], interaction with wildlife [10,11], and by contact with humans [12,13]. AMR bacteria colonizing the animal gastrointestinal tract are shed in feces, thus favoring intra-farm spread and maintenance as well as environmental contamination via farm waste products, including untreated wastewater, sewage sludge, and organic fertilizers such as manure [3,14,15]. Adding to the control of pathogens from outside and inside the farm, biosecurity measures can therefore play a crucial role also in avoiding the selection, maintenance, and spread of AMR microbes within and outside the farm. Accordingly, although the prudent use of antibiotics is key for reducing AMR, adequate farm management practices can also play a fundamental role in containing the AMR burden [4].

With these premises, we investigated a medium-to-large herd where a very high prevalence of ESBL *E. coli* in the feces of calves had been identified in previous unpublished observations. To understand their prevalence, distribution, and antimicrobial resistance traits, as well as to identify possible biosecurity issues, calf feces, cow feces, and the farm and animal environment were investigated by bacteriological culture and microbial sensitivity assays, and a detailed questionnaire was administered to evaluate farming practices.

## 2. Results

### 2.1. Bacteriological Culture Results

The results obtained for all samples collected in the farm are summarized in Table 1.

ESBL *E. coli* was isolated from the feces of 15 of 18 (83.3%) female calves, 13 of 19 (68.4%) male calves, 2 of 3 (66.7%) cows treated for IMI, and waste milk. The feces of the dams of the enrolled calves were all negative. ESBL *E. coli* was also isolated from 8 of 14 (57.14%) environmental samples, including male and female calf pens, the cow feeding rack, the alley floors, the calf drinking water, and the calf feeding buckets.

### 2.2. Antimicrobial Susceptibility Testing of ESBL E. coli

**Figure 1:**
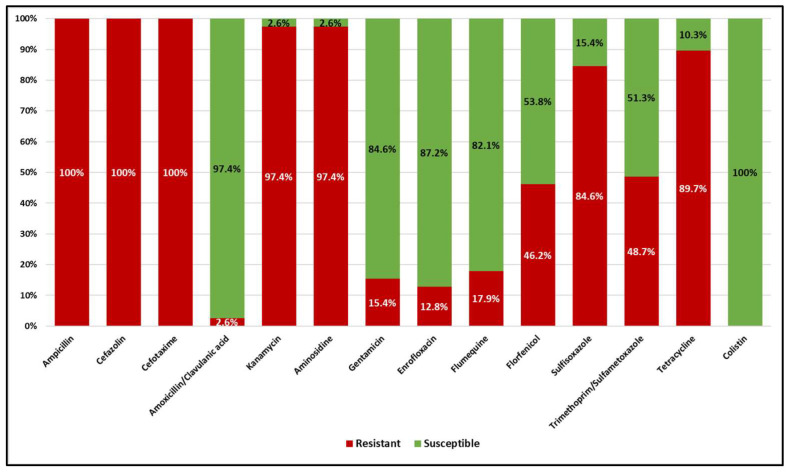
Distribution of resistance and susceptibility of the ESBL *E. coli* isolates to the different antimicrobials according to the plate MIC test.

All *E. coli* isolates were phenotypically positive for the double-disk synergy test (DDST), confirming the production of ESBL. However, all of them were susceptible to the carbapenem class. Based on the MIC results (Figure 1), the highest level of resistance was observed for β-lactams, with all isolates being resistant to ampicillin, cefazolin, and cefotaxime (100%), while only 2.6% of isolates were resistant to amoxicillin/clavulanic acid. Concerning aminoglycosides, 97.4% of isolates were resistant to kanamycin, 97.4% to aminosidine, and 15.4% to gentamicin. Concerning fluoroquinolones, 12.8% were resistant to enrofloxacin and 17.9% to flumequine. Resistance to florfenicol was 46.2%. Concerning sulfonamides, 84.6% were resistant to sulfisoxazole, and 48.7% to trimethoprim/sulfamethoxazole. For the tetracycline class, 89.7% of isolates were resistant. All isolates were susceptible to colistin (100%). Notably, all ESBL *E. coli* isolates were MDR, being resistant to at least three classes of antibiotics. The MIC results are detailed in Supplementary Materials File S1.

Table 2 reports the 12 different ESBL *E. coli* resistance profiles observed in this study. Profile 1 was the most frequent (10 out of 39) and was found in 9 calves (3 male and 6 female calves) and the calf feeding bucket. Profile 2 (8 of 39) was found in 7 calves (4 male and 3 female calves) and waste milk. Profile 3 (6 of 39) was found in 2 out of 3 cows treated for IMI, 3 alley floor samples, and 1 male calf. Profile 4 (4 of 39) was found in 3 female calves and 1 male calf. Profile 5 (3 of 39) was found in 2 female calves and 1 female calf pen. Profile 6 (2 of 39) was found in a male pen and the cow feeding rack. Profiles from 7 to 12 were found only once and in 4 male calves, the calf drinking water, and 1 female calf, respectively.

### 2.3. Hierarchical Clustering of E. coli Isolates Based on the MIC Results

**Figure 2:**
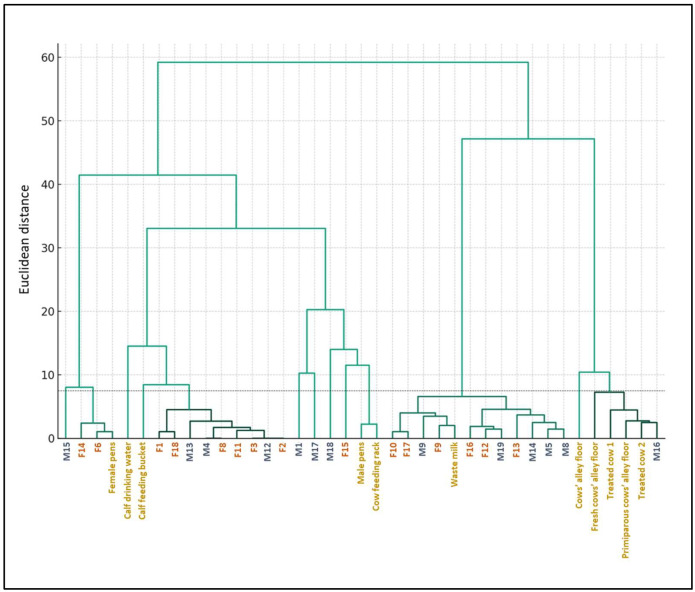
Hierarchical clustering of the ESBL *E. coli* isolates’ MIC profiles based on Ward’s method. Female calves (F) are illustrated in red. Male calves (M) are illustrated in blue. Cows, environment, and water samples are illustrated in gold. The dotted line indicates the significant Euclidean distance cut-off point at 7.5.

Figure 2 illustrates the hierarchical clustering of ESBL *E. coli* isolates based on the MIC test results. Two main branches were observed. One included the isolates from 12 calves, waste milk, treated cows, and alley floors. Within this branch, all the isolates from cows and alley floors and one calf isolate were separated from the waste milk isolate and eleven calf isolates with similar AMR profiles. Another branch included 16 calf isolates and the isolates from calf pens, water and feeding buckets, and the cow feeding rack. Within this branch, one subgroup included the isolates from four calves, male pens, and the cow feeding rack, separated from the isolates from nine calves and the water and feeding buckets, while another subgroup included three calves and the female pen.

Based on the threshold Euclidean distance of 7.5, several statistically significant clusters grouped more than one sample. The largest cluster included 11 calf isolates and the waste milk isolate. The second largest cluster included the isolates from nine calves, closely related to the common feeding bucket and the calf drinking water. The third largest cluster grouped the isolates from one calf, treated cows, and the primiparous and fresh cow alleys, indicating fecal shedding from treated cows as another diffusion route. The fourth one included two female calf isolates and the female calf pens. The other four calf isolates, the male pens, and the cow feeding rack also had similar AMR profiles.

### 2.4. Results of the Biosecurity Questionnaire

The BioCheck.UGent biosecurity questionnaire assessed various aspects of farm management, including general farm organization, sick pen health management and outbreak management, reproduction management, calf pen management and hygiene, calf rearing, herd health management, and milking management. We identified several potential biosecurity issues that might impact ESBL maintenance and diffusion. Concerning calf management and hygiene, cleaning of teat buckets with water without detergents or disinfectants, feeding waste milk to male calves, then sharing buckets between male and female calves, mixed use of calf pens, and occasional cleaning of individual calf pens during the winter season were identified as risk factors. Regarding hygiene issues, lack of pasteurizer cleaning and occasional cleaning of troughs were identified as additional potential risk factors. Finally, the absence of a sick pen was identified as a lack of biosecurity, and the application of dry cow therapy with a first-generation cephalosporin (dihydrated cefalonium) was identified as a factor that could establish a relationship between farm practices and the occurrence and spread of AMR (Supplementary File S2).

## 3. Discussion

This study assessed the presence, distribution, and antimicrobial resistance profiles of ESBL-producing *E. coli* in a medium-sized dairy herd in Northern Italy, hosting nearly 1000 animals, including calves, heifers, and lactating and dry cows. We collected calf and cow feces, waste milk, environmental samples, and water, and we administered a questionnaire to assess the associated risk factors. As a result, most pre-weaned calves, including males and females, carried ESBL *E. coli* in their intestines, and ESBL *E. coli* was also present in the environment and farm equipment in contact with them. Not much is known about the transmission of ESBL *E. coli* among calves and cows, and how it is affected by environmental factors [16]. The hierarchical clustering of ESBL *E. coli* isolates based on the MIC results suggested that multiple MDR strains with different resistance characteristics were circulating in the farm and were found throughout sample types. Different sources and routes could therefore be involved in their dissemination and maintenance, facilitated by incorrect or inadequate management, biosecurity, and hygiene practices.

The herd management interview enabled gathering of information on potential risk factors for the distribution of ESBL microorganisms on the farm. The farmer used waste milk with antibiotic residues for feeding male calves. According to hierarchical clustering based on the isolate MIC profiles, the largest statistically significant cluster included about 40% of all calves’ fecal isolates, the feces of two out of three cows treated for mastitis that contributed to the waste milk, and the waste milk isolate. Among the risk factors associated with the spread of ESBL *E. coli* on cattle farms, the use of waste milk containing antibiotic residues as calf feed appears to play an important role [3,17].

Based on hierarchical clustering, the MIC profiles of the isolates from the calf feeding bucket and drinking water clustered with those of the fecal isolates of over 30% of the calves. Incorrect management practices such as shared or improperly cleaned feeding equipment [3] can favor the diffusion of AMR-carrying bacteria on the farm. Notably, the feeding buckets and calf water buckets were not cleaned with detergents or disinfectants, and sometimes not even rinsed with water between feedings; furthermore, the number of buckets was not adequate for the number of animals on the farm. ESBL *E. coli* was isolated from all these pieces of equipment as well as from the calf drinking water. Moreover, the pasteurizer used to reduce bacterial contamination of milk was not cleaned between cycles. Poor cleaning is one of the factors favoring bacterial contamination and multiplication, leading to higher microbial loads [18]. Indeed, we also isolated ESBL *E. coli* from the farm’s pasteurized waste milk. Moreover, many positive calves were females, which should not have received waste milk. Shared feeding buckets, as well as their unproper cleaning, might also facilitate the transmission of ESBL *E. coli* between male and female calves.

Shared calf pens and their poor hygiene may also play a role in promoting the diffusion of AMR bacteria. We isolated ESBL *E. coli* from both male and female calf pens, and we observed a relationship between the MIC profiles of these isolates and those from calf feces based on hierarchical clustering. As highlighted in an EFSA scientific opinion paper on calf welfare, the level of cleanliness of the areas used for housing calves is a major determinant of their health [19]. Inaccurate cleaning procedures of the single pens or calf hutches may not adequately remove fecal contamination from the walls, leading them to serve as a reservoir [16].

The cows underwent blanket dry cow therapy (BDCT) with a β-lactam, specifically dihydrated cefalonium. Although this practice is not allowed in Italy, some farms are still using it. BDCT has been reported to be linked to a significant increase in ESBL *E. coli* in calf feces during the colostral phase [8].

The MIC profiles of the ESBL *E. coli* isolated from the feces of cows treated for mastitis were similar to those of the isolates from the cows’ alley floors, suggesting fecal shedding. *E. coli* ESBL shedding can vary greatly among individuals [20], and antibiotic treatment for mastitis could play a role in increasing animal colonization, shedding, and subsequent environmental contamination by AMR-carrying bacteria [9]. The farm evaluated in this study did not have a sick pen, and this represents a lack of biosecurity. Early isolation of sick animals is a crucial practice for preventing the spread of pathogenic bacteria and maintaining herd health [21].

ESBL *E. coli* often carries multiple resistance genes for other antimicrobial drugs than β-lactams, leading to MDR [22]. All the isolates obtained in our study, from the calves, their equipment, and the farm environment, were MDR. On the other hand, resistance to colistin, an antibiotic of last resort for humans [23], was not detected, probably because many developed countries, including Italy, have prohibited its usage in food-producing animals. All ESBL *E. coli* isolates were also carbapenem-sensitive. This is also a positive finding, as carbapenemase-producing *E. coli* causes serious human infections. The study by Waade et al. conducted in Germany in 2021 reported similar results since the ESBL-producing isolates were 92.9% *E. coli*, and 60.6% of ESBL-producing isolates were resistant to one or more classes of antibiotics including penicillins and cephalosporins but were sensitive to carbapenems [24].

## 4. Materials and Methods

### 4.1. Farm Description and Ethics Statement

The farm was located in Northern Italy and consisted of 1000 animals of which 450 were lactating Italian Friesian cows. It is accredited free from infectious bovine rhinotracheitis (IBR) and vaccinated for neonatal diarrhea agents and type-1 and type-2 bovine viral diarrhea virus (BVDV). The farm does not use an in-house colostrum bank, but the colostrum is taken by the calves directly from the dam. The farmer used pasteurized waste milk to feed male calves. Based on the questionnaire, waste milk given to the calves was mainly represented by milk with high somatic cell count (SCC) and milk from cows treated with antibiotics. The waste milk produced on the farm at the time of the visit came from animals treated for mastitis with beta-lactam antibiotics (amoxicillin/clavulanic acid). The study was conducted in accordance with the Declaration of Helsinki and approved by the Institutional Committee for Animal Welfare of the University of Milan (protocol number 99_2023).

### 4.2. Questionnaire

A questionnaire was completed together with the herd manager (Supplementary File S2). The questionnaire follows the Biocheck.UGent checklist [25] and was used to assess different aspects of herd management, the use of antibiotics, and farm biosecurity. The questionnaire was also integrated with further aspects based on previous studies on ESBL *E. coli* risk factors [9,17,26]. The form was divided into several sections: I. General questions about farm organization: how many animals are present in the different categories, who works with the animals; II. Health management in the sick pen and management of outbreaks; III. Reproduction management; IV. Calving pen management and hygiene questions; V. Calf rearing: colostrum feeding management, milk feeding management, calf housing, vaccinations, and treatments; VI. Health management of the herd; VII. Milking management.

### 4.3. Animals and Sample Collection

We collected fecal samples from 37 healthy dairy calves (19 males, 18 females) aged 7–21 days. All calves were free from diarrhea and had not been treated with antibiotics. Males were fed waste milk, while females received commercial milk replacer. We sampled the feces of the 26 dams present on the farm (the 9 missing dams had been sold or sent to the slaughterhouse) and of 3 cows treated for intramammary infection (IMI) with amoxicillin/clavulanic acid that contributed to the waste milk. All the fecal samples were collected from the rectal ampoule using gloves, transported to the laboratory in refrigerated conditions, and frozen at −20 °C for two to five days until analysis [27]. Waste milk was collected directly from the pasteurizer and kept refrigerated until arrival at the laboratory. Three calf pens were sampled by rubbing sterile gauzes against the inner wall of the pens over an area of about 150 × 30 cm^2^ at the height of the calves’ noses, avoiding obvious fecal smears, then stored in sterile 50 mL Falcon^®^ tubes. During the sampling process, two separate sterile swabs were used. One swab was rubbed thoroughly against the bottom and inner wall of a calf feeding bucket, while the other swab was used to collect samples from the inside of the nipple. Two water samples of 150 mL were collected into sterile containers from the calf watering buckets and one from the cow watering trough, respectively. Two environmental samples were also taken with gauzes from the cow feeding rack and one from the cow’s berth tube. Disposable fabric socks were used to collect three samples from the barn floors by walking down the alleys one time, and then inserted in sterile plastic bags: one from the cow alley, one from the primiparous cow alley, and one from the fresh cow alley, respectively. All environmental and water samples were stored at refrigerated temperature until arrival at the laboratory.

### 4.4. Isolation and Characterization of ESBL-Producing E. coli

Environmental swabs and feces (0.1 g) were enriched in 5 mL of Müeller Hinton broth (Microbiol, Cagliari, Italy) and incubated at 37 °C under aerobic conditions for 18–24 h. For environmental samples, 30 mL of Müeller Hinton broth was added to the Falcon tubes and plastic bags containing the samples and incubated at 37 °C for 18–24 h. One-hundred milliliters of water was added to an equal amount of double-strength enrichment broth and incubated at 37 °C for 48 h. All the feces and environmental samples were cultured on CHROMagar™ ESBL agar plates (CHROMagar, Paris, France) and MacConkey agar as a control medium (Oxoid Ltd., Basingstoke, UK), and incubated at 37 °C for 18–24 h. Pasteurized waste milk was seeded on blood agar plates (Microbiol, Cagliari, Italy) and CHROMagar™ ESBL agar plates in amounts of 100 μL and incubated at 37 °C for 24 h. Colonies indicating ESBL bacteria grown on CHROMagar™ ESBL agar plates were picked and submitted to species identification with the MBT Microflex LT/SH MALDI-TOF mass spectrometer (Bruker Daltonik GmbH, Bremen, Germany) as described previously [28]. After species identification, the colonies recovered from CHROMagar™ ESBL agar plates were sub-cultured on blood agar plates (Microbiol, Cagliari, Italy) and subjected to ESBL phenotyping assessment using the double-disk synergy test (DDST) to assess carbapenemase production according to the EUCAST guidelines [29].

### 4.5. Antimicrobial Susceptibility Testing

A Sensititre^TM^ ITISVE1 plate (Thermo Fisher Scientific^®^, Waltham, MA, USA) was used to determine the MIC of the antimicrobials commonly used in dairy herds against the ESBL *E. coli* isolates. The plate contained the following antibiotics: flumequine (range 1–16 μg/mL); amoxicillin/clavulanic acid (0.25–32 μg/mL); ampicillin (0.25–32 μg/mL); cefazolin (0.5–8 μg/mL); cefotaxime (0.5–4 μg/mL); sulfisoxazole (128–512 μg/mL); colistin (0.03–8 μg/mL); enrofloxacin (0.02–32 μg/mL); florfenicol (1–64 μg/mL); gentamicin (0.25–32 μg/mL); tetracycline (0.5–16 μg/mL); trimethoprim/sulfamethoxazole (0.06–16 μg/mL); aminosidine (1–32 μg/mL); kanamycin (2–32 μg/mL). Quality control for Sensititre plates was performed using *E. coli* strain ATCC 25922 and the Sensititre™ SWIN™ Software System V. 3.4 (3.4.6.2) (Sensititre^TM^, Thermo Fisher Scientific^®^, Waltham, MA, USA). The MIC results were interpreted according to the manufacturer’s instructions using CLSI VET08 4th edition [30] (V = Vet), CLSI VET06 1st edition [31] (V = Vet), CLSI M100 29th edition (H = Human) [32], EUCAST v.11.0 [33], CASFM 2019 [34]. ESBL *E. coli* isolates resistant to at least 3 classes of antibiotics were classified as MDR [35], and intermediate isolates were classified as susceptible.

### 4.6. Hierarchical Clustering

Non-supervised hierarchical cluster analysis using Ward’s method was performed based on the MIC values [36,37]. A total of 21 parameters were obtained by assigning to each MIC value ranging from >512 μg/mL to ≤0.015625 a number from 1 to 21 according to decreasing antibiotic concentrations. The profiles obtained for each sample after the conversion were used to construct a dendrogram. This technique was chosen for its effectiveness in minimizing variance within the clusters, allowing us to identify groups of isolates with similar resistance patterns. The resulting dendrogram provides a visual representation of the progressive merging of the clusters based on the Euclidean distance. The dendrogram was cut (maximum distance for clustering) at a height of 7.5. This cut-off point was chosen based on statistical significance, ensuring that each cluster represented a distinctive group of isolates with similar characteristics. The analysis was conducted using the SciPy library (version 1.11.4, https://scipy.org/, accessed on 18 January 2024) within the Python environment (version 3.10.12, https://www.python.org/, accessed on 18 January 2024).

## 5. Conclusions

This study highlighted the widespread presence of ESBL *E. coli* in the dairy farm and the relevant presence and circulation of MDR strains in association with different sources and sample types. Prudent antibiotic use remains the most relevant driver enabling the reduction in and control of AMR bacteria. Nevertheless, adherence to good internal and external biosecurity practices, hygiene of facilities and equipment, correct feeding procedures, and correct animal management might also significantly contribute to reducing and controlling AMR bacteria.

## Figures and Tables

**Table 1:** Summary of analyzed samples and respective bacteriological results.

Sample Type	*n*	ESBL *E. coli* (%)
Female calf feces	18	15 (83.3%)
Male calf feces	19	13 (68.4%)
Treated cow feces	3	2 (66%)
Dam feces	26	0
Waste milk	1	1 (100%)
Male calf pens	1	1 (100%)
Female calf pens	1	1 (100%)
Mixed-use calf pens	1	0
Calf feeding bucket	2	1 (50%)
Calf drinking water	2	1 (50%)
Cow alleys	3	3 (100%)
Cow’s berth tube	1	0
Cow water trough	1	0
Cow feeding rack	2	1 (50%)

**Table 2:** Antimicrobial resistance profiles of the ESBL *E. coli* isolates. R, resistant; S, sensitive; MDR, multidrug-resistant. The number of antimicrobial classes is reported in parentheses.

Resistance Profile	Number of Isolates	Aminosidine	Amoxicillin/Clavulanic Acid	Ampicillin	Cefazolin	Cefotaxime	Colistin	Enrofloxacin	Florfenicol	Flumequine	Gentamicin	Kanamycin	Sulfisoxazole	Tetracycline	Trimethoprim/Sulfamethoxazole	MDR (Class Number)
1	10	R	S	R	R	R	S	S	R	S	S	R	R	R	R	(5)
2	8	R	S	R	R	R	S	S	S	S	S	R	R	R	S	(4)
3	6	R	S	R	R	R	S	S	S	S	S	R	S	R	S	(3)
4	4	R	S	R	R	R	S	S	R	S	S	R	R	R	S	(5)
5	3	R	S	R	R	R	S	R	S	R	S	R	R	S	R	(4)
6	2	R	S	R	R	R	S	S	R	S	R	R	R	R	R	(5)
7	1	R	S	R	R	R	S	S	S	R	R	R	R	R	S	(5)
8	1	S	S	R	R	R	S	S	S	S	S	S	R	R	R	(3)
9	1	R	S	R	R	R	S	S	S	R	R	R	R	S	R	(4)
10	1	R	S	R	R	R	S	R	S	R	S	R	R	R	R	(5)
11	1	R	S	R	R	R	S	S	R	S	R	R	R	R	S	(5)
12	1	R	R	R	R	R	S	R	R	R	R	R	R	R	R	(6)

## Data Availability

All pertinent data generated in this study are provided in the Supplementary Materials.

## Supplementary Materials

The following supporting information can be downloaded at: https://www.mdpi.com/article/10.3390/antibiotics13030241/s1, Supplementary File S1: MIC values expressed in µg/mL for the ESBL *E. coli* isolates assessed in this study with the plate assay. Supplementary File S2: Questionnaire of the study Multidrug-resistant extended-spectrum beta-lactamase (ESBL)-producing *Escherichia coli* in a dairy herd: distribution and antimicrobial resistance profiles.
