# Supplementary material for: Multidrug-Resistant Extended-Spectrum Beta-Lactamase (ESBL)-Producing Escherichia coli in a Dairy Herd: Distribution and Antimicrobial Resistance Profiles
Source: Antibiotics (Basel). 2024 Mar 5;13(3):241. doi: 10.3390/antibiotics13030241 (PMC10967312; doi:10.3390/antibiotics13030241)
Supplement: Supplementary file 1 [file antibiotics-13-00241-s001.zip › antibiotics-2874511-supplementary/Supplementary File S1.pdf]

Supplementary File S1. MIC values expressed in μg/mL for the ESBL *E. coli* isolates assessed with the plate assay.

**Isolate**	**Aminosidine**	**Amoxicillin/Clavulanic Acid**	**Ampicillin**	**Cefazolin**	**Cefotaxime**	**Colistin**	**Enrofloxacin**	**Florfenicol**	**Flumequine**	**Gentamicin**	**Kanamycin**	**Sulfisoxazole**	**Tetracyclin**	**Trimethoprim/Sulfametoxazole**
M1	>32	=8	>32	>8	>4	=0.25	=0.5	=8	=16	>32	>32	>512	>16	=0.125
M4	>32	=8	>32	>8	>4	=0.25	=0.031	>64	<=1	=1	>32	>512	>16	>16
M5	>32	=8	>32	>8	>4	=0.25	<=0.016	=8	<=1	=2	>32	>512	>16	=0.125
M8	>32	=8	>32	>8	>4	=0.25	=0.031	=8	<=1	=1	>32	>512	>16	=0.125
M9	>32	=8	>32	>8	>4	=0.25	=0.063	=8	<=1	=0.5	>32	>512	>16	=0.25
M12	>32	=8	>32	>8	>4	=0.5	=0.031	>64	<=1	=1	>32	>512	>16	>16
M13	>32	=16	>32	>8	>4	=0.5	=0.031	>64	<=1	=1	>32	>512	>16	>16
M14	>32	=8	>32	>8	>4	=0.25	=0.031	=8	<=1	=2	>32	>512	>16	=0.25
M15	>32	=8	>32	>8	>4	=0.25	=32	=4	>16	=0.5	>32	>512	>16	>16
M16	>32	=8	>32	>8	>4	=0.25	=0.25	=4	=8	=1	>32	<=128	>16	<=0.063
M17	>32	=8	>32	>8	>4	=0.25	=0.063	>64	<=1	>32	>32	>512	>16	=0.25
M18	>32	=32	>32	>8	>4	=0.5	=32	>64	>16	>32	>32	>512	>16	>16
M19	>32	=8	>32	>8	>4	=0.25	=0.031	=16	<=1	=1	>32	>512	>16	=0.25
F1	>32	=8	>32	>8	>4	=0.5	=0.063	>64	<=1	=1	>32	>512	>16	>16
F2	>32	=8	>32	>8	>4	=0.5	=0.031	>64	<=1	=1	>32	>512	>16	>16
F3	>32	=8	>32	>8	>4	=0.5	=0.031	>64	<=1	=1	>32	>512	>16	>16
F6	>32	=8	>32	>8	>4	=0.5	=32	=8	>16	=1	>32	>512	=2	>16
F8	>32	=8	>32	>8	>4	=0.25	=0.031	>64	<=1	=1	>32	>512	>16	>16
F9	>32	=8	>32	>8	>4	=0.25	=0.031	=8	<=1	=0.5	>32	>512	>16	=0.125

Isolate	Aminosidine	Amoxicillin/Clavulanic Acid	Ampicillin	Cefazolin	Cefotaxime	Colistin	Enrofloxacin	Florfenicol	Flumequine	Gentamicin	Kanamycin	Sulfisoxazole	Tetracyclin	Trimethoprim/Sulfametoxazole
F10	>32	=8	>32	>8	>4	=0.25	<=0.016	=8	<=1	=0.5	>32	>512	>16	=0.5
F11	>32	=8	>32	>8	>4	=0.5	=0.031	>64	<=1	=2	>32	>512	>16	>16
F12	>32	=8	>32	>8	>4	=0.5	=0.031	=16	<=1	=2	>32	>512	>16	=0.25
F13	>32	=16	>32	>8	>4	=0.25	=0.031	=16	<=1	=1	>32	>512	>16	=0.125
F14	>32	=8	>32	>8	>4	=0.5	=32	=8	>16	=0.5	>32	>512	=2	>16
F15	>32	=8	>32	>8	>4	=0.25	=0.5	=8	=16	=32	>32	>512	=2	>16
F16	>32	=8	>32	>8	>4	=0.25	<=0.016	=16	<=1	=1	>32	>512	>16	=0.5
F17	>32	=8	>32	>8	>4	=0.25	<=0.016	=8	<=1	=0.5	>32	>512	>16	=0.25
F18	>32	=8	>32	>8	>4	=0.5	=0.125	>64	<=1	=1	>32	>512	>16	> 16
Male pens	>32	=8	>32	>8	>4	=0.5	=1	>64	=2	>32	>32	>512	>16	>16
Female pens	>32	=8	>32	>8	>4	=0.5	>32	=8	>16	=1	>32	>512	= 2	>16
Treated cow 1	>32	=8	>32	>8	>4	=0.5	=0.5	=8	=8	=1	>32	<=128	>16	<=0.063
Treated cow 2	>32	=8	>32	>8	>4	=0.5	=1	=8	=4	=0.5	>32	<=128	>16	<=0.063
Waste milk	>32	=16	>32	>8	>4	=0.25	=0.031	=8	<=1	=0.5	>32	>512	>16	=0.125
Cow feeding rack	>32	=8	>32	>8	>4	=0.25	=0.5	>64	=2	>32	>32	>512	>16	>16
Cow alley floor	>32	=8	>32	>8	>4	=0.25	=0.031	=8	<=1	=0.5	>32	<=128	>16	<=0.063
Fresh cow alley floor	>32	=8	>32	>8	>4	=0.25	=1	=8	=4	=1	>32	<=128	>16	=0.5
Primiparous cow alley floor	>32	=8	>32	>8	>4	=0.5	=0.5	=4	=8	=0.5	>32	<=128	>16	<=0.063
Calf feeding bucket	>32	=8	>32	>8	>4	=0.5	=0.5	>64	=4	=2	>32	>512	>16	>16
Calf drinking water	=4	=16	>32	>8	>4	=0.25	=0.5	=8	=2	=1	=4	>512	>16	>16

Values are expressed in μg/mL.
