# Supplementary material for: Multidrug-Resistant Extended-Spectrum Beta-Lactamase (ESBL)-Producing Escherichia coli in a Dairy Herd: Distribution and Antimicrobial Resistance Profiles
Source: Antibiotics (Basel). 2024 Mar 5;13(3):241. doi: 10.3390/antibiotics13030241 (PMC10967312; doi:10.3390/antibiotics13030241)
Supplement: Supplementary file 1 [file antibiotics-13-00241-s001.zip › antibiotics-2874511-supplementary/Supplementary File S2.pdf]

Supplementary File S2. Questionnaire of the study Multidrug-Resistant Extended-Spectrum Betalactamase (ESBL)-Producing *Escherichia Coli* in a Dairy Herd: Distribution and Antimicrobial Resistance Profiles.

Data of the participants

____

____

I: General questions: breeding organization

Who takes care of the cattle?

X The owner of the herd

□ The family member(s)

□ The employee(s)

□ The contractor(s)

How many… are present on the farm?

Breeding bulls	Dairy cows	Dry cows	Heifers	Weaned calves	Suckling calves
**0**	**457**	**86**	**254**	**153**	**50**

How many milking groups are there, and how are they divided (fresh/fresh/tight)?
4 groups (primiparous - early - mid-late stage cows)

Do you buy animals? No

Upon entering the farm, do you have to put…?

	Always	Sometimes	Never
Specific clothing	○	○	**x**
Single use clothing	**x**	○	○

<u>II: Health management in the sick pen and management outbreaks</u>

Are sick animals physically isolated from the rest of the herd?

x Always

○ Sometimes

○ Never

Are there specific equipment and materials (e.g., buckets, thermometers, cleaning and feeding utensils, gastric probes...) in the area used for sick animals?

x Yes

○ No

Is this specific equipment cleaned and disinfected before a new animal enters the sick animal area?

○ It is cleaned and disinfected

x It is only cleaned

○ No

Are cattle in the area used for sick animals wholly separated from the rest of the herd?

○ Yes

x No

I... before entering the area used for sick cattle

	Always	Sometimes	Never
Use specific boots	○	○	**x**
Wear specific clothing	**x**	○	○
Wash hands/use gloves	○	**x**	○

Is the area used for sick animals empty after each use?

○ Yes

x No

The area used for sick animals is…before each new introduction of other sick animals

	Always	Sometimes	Never
Cleaned	**x**	○	○
Disinfected	**x**	○	○
Dried	**x**	○	○

Do you care for sick livestock before or after healthy livestock?

○ Before

○ After

x No specific order

Can a group of sick cattle be separated from the rest of the herd in case of an epidemic?

○ Always

○ Sometimes

x Never

Is a record kept of animal health data?

x Yes

○ No

Are there written protocols for vaccination, disease treatment, and hygiene procedures?

○ Yes

x No

Is a bacteriological examination of the udder of all cows performed regularly (i.e., at least once a year)?

x Yes

○ No

What happens to animals identified as carriers of disease?

□ Elimination (always)	**x** Elimination (sometimes).
□ Segregation (always)	□ Segregation (sometimes)
□ Nothing (always)	□ Nothing (sometimes)
□ Delayed elimination (always)	□ Delayed elimination (sometimes)

Litter type used in the sick pen: Straw

III: Reproduction management

Are calving boxes available on the farm?

x Yes, there are individual calving boxes

○ Yes, there are shared calving boxes

○ No

Has the calving box ever been used to house sick animals, or is it adjacent to areas used for that?

○ Always

x Sometimes

○ Never

Are the cows in the calving box completely separated from the other animals?

○ Yes

x No

Are... in the calving box?

	Always	Sometimes	Never
**Compartment-specific boots worn**	○	○	**x**
**Compartment-specific clothing worn**	**x**	○	○
**Hands washed/(new) gloves used**	**x**	○	○

The calving box is ... before the introduction of new animals?

	Always	Sometimes	Never
Cleaned	○	**x**	○
Disinfected	○	○	**x**
Dried	○	○	**x**

When helping with deliveries/abortions, are the hands and obstetric materials used always cleaned and disinfected before and after each delivery/abortion?

x They are cleaned and disinfected

○ They are only cleaned

○ No

When does the separation of the calf from the mother take place?

○ Within one hour of the calf's birth

x No separation; the calf remains with the mother as a suckling calf

○ Just after birth

If an abortion occurs, is the cow tested (i.e., abortion protocol)?

○ Always

x Sometimes

○ Never

Where are fetal tissues and membranes disposed of after a birth/abortion?

○ Are they left in the barn/delivery area or where the abortion occurred

○ They are eaten by the cow/dog/other

x They are put on the manure pile/slurry pit

○ Other

Type of bedding used and how often changed: Straw, changed when necessary (evaluation of dirtiness)

IV: Calf rearing

How many colostrum meals do they make? 3: The first with the mother, one or two the day after

How many milk meals do they take? Two each day

How many liters of colostrum are given to the calf in the first six hours after birth? Ad libitum

How is the colostrum administered?

x Directly from the mother

○ Bottle

x Bucket

○ Probe

Does the mother give colostrum, or is the colostrum bank used on the farm?

There is no colostrum bank, and it is given by the mother.

Is it checked whether the quality of the colostrum is sufficient?

x Yes and method: refractometer

○ No

Is there a frozen or artificial supply of colostrum in case the mother does not provide enough milk, or the colostrum has insufficient quality?

x No

○ Yes, there is a frozen supply from other cows on my farm

○ Yes, there is a frozen reserve from outside my farm

○ Yes, there is an artificial reserve

If colostrum is not fed to the calf immediately after milking, is the colostrum stored in a refrigerator?

○ Yes

x No

Are the materials used for colostrum administration (e.g., test tubes, bottles, etc.) cleaned and disinfected after each use?

○ They are cleaned and disinfected

x They are only cleaned

○ No

Are calves fed waste milk (i.e., milk not suitable for the bulk tank milk)?

x Always (only the male calves)

○ Sometimes (specify cases)

○ Never

If they give waste milk, what type is it?

x High cells

x Antibiotic

○ Colostrum/transitional milk

Is the waste milk pasteurized? If yes, according to what process?

Yes, at 65 °C for 30 minutes (after that, it remains for 12 hours and heat when needed at 40°C).

If pasteurizer is used, does it undergo washing processes?

Yes, only with water – no disinfectant

To whom is the waste milk fed, and for how long?

To the male calves until they are sold

List the division of calf groups from birth to weaning

Males and females in single pens; females in boxes with the calf feeder machine.

V: Calf housing

Are calves housed in individual pens/boxes or separate areas?

x Yes

○ No

Are individual pens/calf boxes/separate areas empty after each use?

x Yes

○ No

Are the individual pens/calf stalls/separated areas … before each new introduction of calves?

	Always	Sometimes	Never
Cleaned	**x**	○	○
Disinfected	○	**x**	○
Dried	○	○	○

Is calf contact between different group pens/single stalls/separate areas possible?

x Yes

○ No

How large (number of animals) are the groups of calves that are grouped from individual pens/separated areas to group pens? 20>40

Are the group pens empty after each use?

○ Yes

x No

Is the group enclosure (including single pens) ... before each new introduction of calves?

	Always	Sometimes	Never
Cleaned	○	**x**	○
Disinfected	○	**x**	○
Dried	○	○	**x**

Are the single pens disinfected?

Single pens are disinfected only in the spring/summer/autumn period but not in winter.

During the same feeding session, are milk-feeding buckets/teats reused between calves?

x Yes

○ No

Are the feeding buckets cleaned after each feeding session?

○ Yes

x No

Is there a feeding order for calves?

Male calves are fed first, then females from the same buckets without washing them.

<u>**VI: Health management of the herd**</u>

Do you use selective dry cow therapy? No, all the cows receive blanket dry cow therapy with cefalonium dihydrate**.**

Mastitis predominantly pathogens: Environmental pathogens

What antibiotics do you use for mastitis? Amoxicillin/clavulanic acid

Which antibiotics do you use to treat diarrheic calves? Gentamicin in the case of *E. coli*

Microbiological fecal control of calves with diarrhea?

○ Yes (how many times/year______________________)

x No

Do you perform Cryptosporidium spp. prevention? No

Do you vaccinate dry cows for neonatal calf diarrhea?

x Yes: Rota-Corona

○ No

Do you vaccinate calf for bronchopneumonia?

○ Yes

x No

Do you vaccinate for BVD?

x Yes

○ No

IBR herd status:

x IBR free

○ IBR vaccination

○ Unknown

Is the drinking water quality checked annually at the source or storage tank by bacteriological analysis?

x Yes

○ No, but I am using a water supply

○ No

Is the drinking water quality checked annually at the main distribution points (watering source for livestock) by bacteriological analysis?

x Yes

○ No

How often do you change the water in the calf buckets?

○ Always

x Sometimes (daily or every two days)

○ Never

Are the cow water troughs cleaned?

○ Always

x Sometimes

○ Never

VII: Milking management

Do you use a milking parlor or a milking robot?

○ Milking robot

x Milking parlor

How many times/year is performed a static measurement of the milking equipment? 2

How many cows are milked on average? 380

How many milkings are performed per day? 2

How many milking positions are in the milking parlor? 24 + 24

Do you use rubber or silicon liner liners?

x Rubber liners

○ Silicon liners

Are milking clusters disinfected between cows?

x Yes, after each cow

○ Yes, after cows with mastitis

○ Yes, after cows with high somatic cell count (SCC)

○ No

How are milking clusters disinfected between animals?

○ With hot water (more than 75°C)

○ With steam

x Other

Are teats cleaned before milking? If yes, how?

○ Yes, pre-dipping

○ Yes, dry cleaning with separate towels

○ Yes, wet washing and drying afterward with separate towels

x Yes, wet washing, but not drying afterward (disposable disinfectant wipes)

○ No

Is the first milk examined during preliminary extraction?

x Yes

○ No

Are the teats disinfected after being milked?

x Yes, with dipping

○ Yes, with a spray

○ No

Are cows kept at the station for a while after milking?

○ Yes, from 30 minutes to one hour

○ Yes, for more than one hour

x No

Are the cows milked in a specific order?

x Yes, cows with mastitis and/or high SCC are milked last

○ Yes, cows with mastitis and/or high SCC are milked first

○ No

Are the udders of lactating cows shaved?

○ Yes, two or more per year

○ Yes, once a year

x No

Are the tails of lactating cows shaved?

○ Yes, two or more times a year

○ Yes, once a year

x No

What type of bedding do you use for lactating cows? Straw in the cattle cubicle
